# Gestational Diabetes Mellitus and Risk of Childhood Overweight and Obesity in Offspring: A Systematic Review

**DOI:** 10.1155/2011/541308

**Published:** 2011-09-22

**Authors:** Shin Y. Kim, J. Lucinda England, J. Andrea Sharma, Terry Njoroge

**Affiliations:** Division of Reproductive Health, National Center for Chronic Disease Prevention and Health Promotion, Centers for Disease Control and Prevention, Atlanta, GA 30341, USA

## Abstract

We systematically reviewed research examining the association between gestational diabetes (GDM) and childhood overweight and obesity. We identified studies from three sources: (1) a PubMed search of articles published between January 1990–January 2011, (2) reference lists of publications from the PubMed search, and (3) reference lists of review articles. We included studies that examined GDM separately from pregestational diabetes and childhood overweight or obesity defined as BMI > 85th or 95th percentile. A total of 12 studies were included in the systematic review. Crude odds ratios for the relationship between GDM and childhood overweight or obesity ranged from 0.7 to 6.3; in 8 studies, the associations were not statistically significant. In only 3 studies were results adjusted for any confounders; in the 2 that adjusted for prepregnancy obesity, the GDM and childhood overweight or obesity associations were attenuated and not statistically significant after adjustment. This paper demonstrates inconsistent evidence of an association between GDM and offspring overweight and obesity due to the methodological limitations of existing studies. Recommendations for future research are presented, which address methodological challenges.

## 1. Introduction

Approximately 4–6% of pregnancies in the United States are complicated by diabetes mellitus (DM), making it one of the most common serious medical complications of pregnancy [1–5]. The majority of cases (>80%) are diagnosed for the first time during pregnancy (gestational DM); the remaining cases are pregestational DM (type 1 or type 2) [2]. Furthermore, the prevalence of diabetes in pregnancy is increasing in the United States, concurrent with the rising prevalence of obesity and type 2 diabetes in the general population; this increase is not explained by changes in the prevalence of other known maternal risk factors, such as advanced maternal age [6–8]. 

It is commonly stated that intrauterine exposure to maternal diabetes places offspring at increased risk for long-term adverse outcomes including overweight and obesity. In addition, it has been suggested that infants of women with diabetes should be specifically targeted for obesity prevention interventions. This suggestion raises several issues. Maternal obesity is also a risk factor for offspring overweight and obesity, is associated with maternal diabetes, and is a more prevalent condition than either gestational or pregestational DM [9–12]. It is unclear, therefore, whether it would be most effective to target infants of women with diabetes specifically, or all infants of women with prepregnancy obesity. Second, studies commonly cited as providing support for a causal association between maternal gestational DM and offspring overweight and obesity, including those conducted among Pima Indian women, have design limitations including combining pregestational and gestational DM into one exposure group, examination of exposed individuals only without a nondiabetic control group, and failure to control for important potential confounders [13–15]. 

In this study, we systematically reviewed studies examining the association between gestational DM and childhood overweight and obesity. We focused on gestational DM because it is the most prevalent form of diabetes in pregnancy. We summarized findings and addressed methodological limitations of previously published studies. We did not calculate a combined single estimate of the findings due to the heterogeneity of methodology and rigor among the studies. Finally, we provided recommendations regarding approaches for future studies.

## 2. Materials and Methods

### 2.1. Search Process

We used recommendations from the Meta-analysis of Observational Studies in Epidemiology (MOOSE) guidelines to identify studies for possible inclusion in this analysis [16]. We first searched PubMed records for the period January 1990–January 2011 using the following search terms for childhood overweight/obesity: (Pregn*) AND (GDM OR gestational diabetes OR diabetes OR glucose) AND (Overweight or obes* or BMI or body mass index or weight gain) AND (child* OR adolescen* OR offspring OR long term OR fetal program* OR imprint*). From this search, the full text was retrieved for papers in which the abstracts mentioned a relationship between maternal gestational DM and childhood overweight and obesity. Studies that did not have full text in English were translated for review. 

Next, we manually reviewed the reference lists of the publications retrieved and obtained the entire text of publications that potentially could be included in the systematic review. Finally, we searched the reference lists of review articles on gestational DM and childhood overweight and obesity published in the last five years from January 2006 to January 2011 for additional potential publications. We did not attempt to locate any unpublished studies.

Studies that were considered potentially eligible were then reviewed for inclusion in the analysis based on the following criteria.

1. Data were reported from a cohort with a nondiabetic exposure group or from a case-control study, and not from a case series.
2. Cases of gestational DM were not combined with cases of pregestational DM. We did not exclude studies based on criteria for defining gestational DM.
3. An association was reported (negative, positive, or null) for childhood (ages 2–18 years) overweight and/or obesity using a BMI-for-age-and-sex of >85 or >95th percentile. We did not exclude studies based on the authors' reference population for child growth. However, we did exclude studies that examined BMI only as a continuous variable.
4. The manuscript was written in English or in a language that could be translated with resources available at CDC.
5. For studies with offspring outcome assessment at multiple ages, findings from data for longest duration of followup were used. When multiple articles from the same study population met the study criteria, we included only the publication with the most recent data available.

### 2.2. Data Abstraction

All articles were read and reviewed by two authors (S. Y. Kim and T. Njoroge). The authors abstracted the design, setting, location, and time period; the number and characteristics of study participants; the gestational DM diagnosis criteria; the source(s) for childhood obesity (e.g., medical records and clinical databases), and the statistical methods.

### 2.3. Statistical Methods

For each study, we constructed separate two-by-two tables to calculate unadjusted odds ratios (ORs) and 95% confidence intervals (CIs) of gestational DM and each childhood weight outcome analyzed by the study's authors. If a study presented only prevalence estimates, we contacted the author to request the actual sample size for the percentages. We also presented the adjusted odds ratios when available.

## 3. Results

**Childhood Obesity**

**Figure 1:**
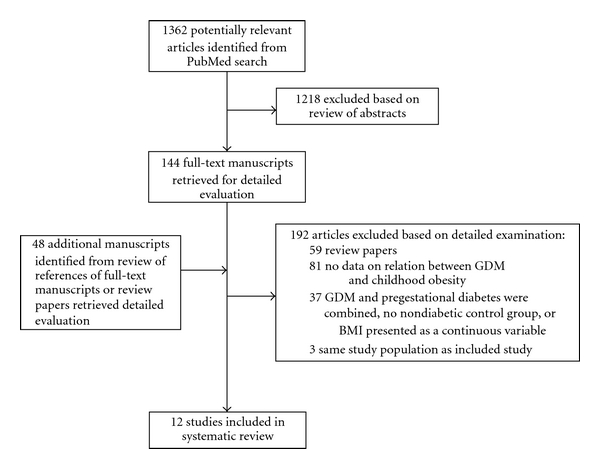
Flow diagram showing the number of studies included in and excluded from the systematic review for childhood obesity.

We identified 1362 potentially relevant studies by searching PubMed; of these, 144 abstracts reported a finding on the relationship between maternal gestational DM and childhood obesity and the full texts of these articles were retrieved for detailed examination (Figure 1). We also reviewed the reference lists of all 144 studies, and identified an additional 48 studies for possible inclusion. After review of these 192 articles, 140 were excluded, either because they did not report the results of a cohort or case-control study or because they clearly did not address an association between maternal gestational DM and childhood obesity; thus, 52 studies were considered further for inclusion. Of these, 37 studies were excluded because gestational and pregestational DM were combined, there was no nondiabetic control group, or BMI was not presented as 85th or 95th percentile or it was presented only as a continuous variable. An additional 3 studies were excluded because later papers reported results on the same study population. A total of 12 studies met the inclusion criteria [17–28].

Six of the studies were conducted in the United States, and one each was conducted in Germany, Brazil, the United Kingdom, Hong Kong, Finland, or Poland (Table 1). We translated one study and contacted three authors. No studies were excluded because of lack of language translation resources. Sources of information on maternal gestational DM diagnosis included maternal self-report, clinical records, or blood glucose measurements. Information used to determine childhood obesity was obtained from parental report, anthropometric measurements obtained as part of a study protocol, hospital records, or clinical databases.

**Figure 2:**
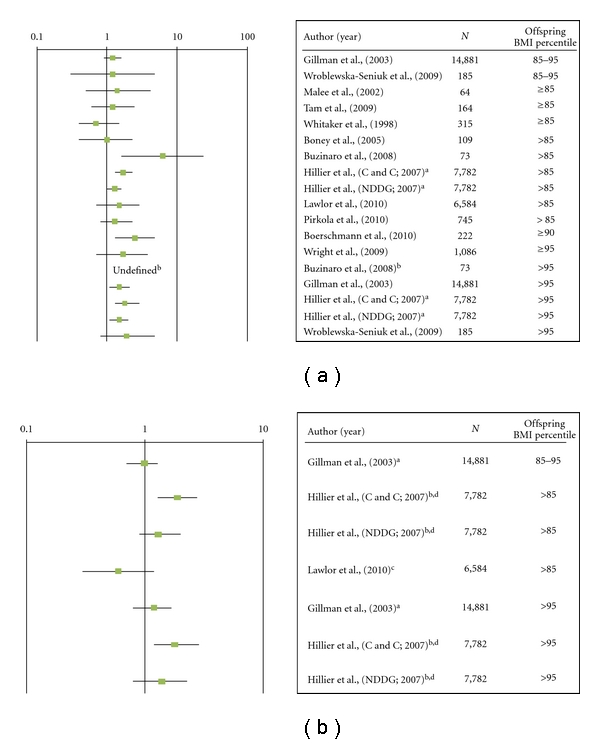
(a) Association of GDM and childhood overweight or obesity, unadjusted odds ratio, and 95% confidence interval. ^a^At the time this analysis was conducted, Kaiser Permanente used the NDDG criteria to diagnose and treat GDM. However, in the analysis, they calculated GDM using both criteria. Therefore, those meeting the NDDG criteria in this analysis were likely treated with diet or diet/insulin, but those meeting only the Carter and Coustan criteria were likely to not be treated. ^b^Undefined because odds ratio could not be calculated with a zero cell. (b) Association of GDM and childhood overweight or obesity compared to a nondiabetic control group among studies that adjusted for any confounders, adjusted odds ratios, and 95% confidence intervals. ^a^Adjusted for maternal BMI and child's age, gender, Tanner stage, TV watching, physical activity, energy intake, birth weight, breastfeeding duration, birth order, and mom's household income, mother's smoking, dietary restraint, weight cycling, weight concerns, and mother's current BMI. ^b^Adjusted for maternal age, parity, weight gain during pregnancy, ethnicity, macrosomia at birth, and sex of child. ^c^Adjusted for maternal prepregnancy BMI and sex, age at outcome, height, height squared, maternal age, social class, parity, smoking during pregnancy, mode of delivery, and maternal prepregnancy BMI. ^d^At the time this analysis was conducted, Kaiser Permanente used the NDDG criteria to diagnose and treat GDM. However, in the analysis, they calculated GDM using both criteria. Therefore, those meeting the NDDG criteria in this analysis were likely treated with diet or diet/insulin, but those meeting only the Carter and Coustan criteria were likely to not be treated.

The crude odds ratios for the relationship between gestational DM and childhood overweight or obesity ranged from 0.7 to 6.3 (Figure 2(a)). When the studies were ordered from offspring overweight to obesity, the magnitude of the association did not increase with increasing levels of offspring BMI. If we exclude the lower and upper values, the OR ranges from 1.0 to 2.5. Eight of the twelve studies had significant findings in the crude analysis. In the three of the twelve studies (two of which were among the eight with a significant finding), the authors adjusted for potential confounders when examining the association between gestational DM and childhood obesity compared to a nondiabetic control group (Figure 2(b)). In the first of the three studies, Gillman and colleagues reported a crude odds ratio (OR) of 1.2 (0.9–1.5) and 1.4 (1.1–2.0) for adolescent weight at 85th–95th percentile and >95th percentile, respectively at age 9–14 years old among the offspring of mothers with gestational DM versus no diabetes [19]. These estimates did not change after adjustment for level of child's physical activity, hours per week watching television, and energy intake. However, adjustment for maternal prepregnancy BMI attenuated the associations for both weight at 85th–95th percentile (AOR = 1.0, 95% CI 0.7–1.3) and at >95th percentile (AOR = 1.2, 95% CI 0.8–1.7), and results were no longer significant. In the second study, Lawlor and colleagues reported a crude OR of 1.5 (0.8, 2.9) for weight >85th percentile at age 9–11 years among offspring of mothers with gestational DM, but this positive finding was reversed after adjustment for prepregnancy BMI (AOR = 0.62, 95% CI 0.3, 1.23) [26]. However, the finding was not significant either before or after the adjustment. In the third study, by Hillier and colleagues, the authors compared treated and nontreated women with gestational DM each to women with no diabetes [20]. They did not adjust for prepregnancy BMI but did adjust for maternal age, parity, gestational weight gain, ethnicity, macrosomia, and sex of child. They reported an adjusted OR of 1.9 (1.3, 2.8) for child's weight >85th percentile and 1.8 (1.1, 2.9) for child's weight >95th percentile at age 5–7 years among offspring of mothers likely not treated for gestational DM (those who met criteria for gestational DM using Carpenter and Coustan criteria only and did not meet the National Diabetes Data Group (NDDG) criteria) compared to offspring of women with no diabetes. They found no significant associations between gestational diabetes and childhood weight >85th percentile (adjusted OR = 1.3 (0.8, 1.9) or for child's weight >95th percentile (adjusted OR = 1.38 (0.8, 2.3)) in women likely treated for gestational DM (those who met criteria for gestational DM using the NDDG criteria). In addition, the authors observed a dose-response relationship between maternal glucose concentration in quartiles among women with no diabetes and risk of childhood overweight and obesity, suggesting that elevated maternal blood glucose does affect risk of overweight and obesity in offspring, and that this risk may be reduced with treatment of gestational DM during pregnancy.

In one of the 12 studies, Boerschmann and colleagues did not adjust for any confounders; however, the authors did present results stratified by maternal BMI among offspring of women with gestational DM. They found that among offspring of women with gestational DM, there was an increase in offspring BMI percentile ≥90 as maternal BMI increased. This increase further supports the strength of maternal BMI [27]. None of the 12 studies included information on genetic factors.

## 4. Discussion

To our knowledge, ours is the first study to systematically review all published case-control and cohort studies specifically examining the association between maternal gestational DM and the prevalence of offspring overweight and obesity. We excluded many studies from our review because of methodological limitations, including the use of an exposure group combining pregestational and gestational DM, and the lack of a nondiabetic control group. We found that studies of the effects of maternal gestational DM on offspring overweight and obesity have yielded inconclusive results, which is consistent with a recent review examining maternal diabetes and offspring BMI z-scores, where they found the association between maternal diabetes and offspring BMI to be no longer significant after adjustment for prepregnancy BMI [29].

## 5. Fetal Exposure to Maternal Hyperglycemia

Women with preexisting diabetes have a substantially increased risk of pregnancy complications including fetal loss, perinatal mortality, and of delivering an infant with congenital anomalies [30]. Although the mechanisms underlying these associations are not completely understood, it has been shown that tight glycemic control in early pregnancy reduces the prevalence of such pregnancy complications [31]. In contrast to women with pregestational DM who are hyperglycemic throughout pregnancy, women with gestational DM typically develop hyperglycemia in the second or third trimester of pregnancy, and pregnancy complications associated with gestational DM are not as severe [32, 33]. Therefore, we cannot assume that pregestational DM and gestational DM have the same effects on fetal development and long-term offspring outcome, and it is important to study associations between these two conditions and offspring outcomes separately. 

In addition to timing of exposure, it is important to consider differences in diagnostic cutpoints for gestational DM. Depending on the cutpoints, it may result in the inclusion of women with milder disease in the gestational DM exposed group, which could attenuate the association between gestational DM and offspring overweight and obesity. Therefore, the severity of disease would be important to consider when examining offspring overweight and obesity.

## 6. Confounders

### 6.1. Maternal Prepregnancy Obesity

Studying the relationships between pregestational and gestational DM and risk of overweight and obesity in the offspring is complicated by difficulties in fully controlling for potential confounders, including maternal obesity, genetic factors, and maternal and infant lifestyle. In our review, we found that while many authors reported positive associations between gestational DM and childhood obesity in crude analysis, their results were not adjusted for important potential confounders, most notably maternal obesity. In the three studies that included adjusted analysis, the associations were attenuated after adjustment for confounders. Furthermore, results were adjusted for maternal obesity in only two studies; in both, associations between gestational DM and childhood overweight or obesity were not significant after adjustment. 

Maternal obesity is an important risk factor for gestational DM; women who are overweight, obese, or severely obese before pregnancy are two-, four-, and eight-times more likely to develop gestational DM compared with normal-weight women [11]. Infants born to overweight and obese mothers are more likely to be macrosomic, even in the absence of gestational diabetes [34]. In a recent study by Catalano et al., the authors found that prepregnancy obesity was significantly associated with offspring being in the highest BMI tertile and for having metabolic dysregulation at age 6–11 years [35]. They also found that prepregnancy obesity was the strongest perinatal predictor of high BMI in childhood, stronger than either maternal glucose homeostasis or weight gain during pregnancy. Although this study was not included in our review because the outcomes were not defined as overweight or obesity, the results provide additional support that gestational DM cannot be evaluated as an independent risk factor for childhood overweight and obesity without accounting for potential confounding from maternal obesity. This concept is further supported by a follow-up study of offspring of women who participated in the Hyperglycemia and Adverse Pregnancy Outcome Study in Belfast, Northern Ireland. The authors found that at age two the overall correlations between maternal glucose during pregnancy and BMI z-score were weak and that birth weight and maternal BMI remained independent predictors of BMI z-score after adjustment [36].

### 6.2. Other Potential Confounders

Other potentially important confounders in the association between gestational DM and offspring overweight and obesity include common social, environmental, and genetic factors shared by both the mothers and the offspring. Poor dietary habits and a sedentary lifestyle in the mother may contribute to increased prepregnancy weight and weight gain during pregnancy, and may increase the risk of poor diet and sedentary behavior for the offspring [10]. However, only one of the studies in our review examined the association between physical activity and nutrition and childhood overweight and obesity [19]. Genetic influence is also important to consider. However, it is difficult to fully control for genetics outside of sibling studies, and none of the studies in our review included genetic factors as potential confounders. To our knowledge, no studies examining associations between gestational DM and offspring obesity have taken all of potentially important confounders, including maternal obesity, gestational weight gain, and maternal and infant lifestyle and genetic factors into account.

## 7. Treatment of Gestational DM

If treatment of gestational DM reduces risk of overweight or obesity in offspring, it may be difficult to consistently detect an association in observational studies. Women with gestational DM are typically instructed to monitor blood sugar and adjust their dietary habits, which may improve long-term offspring outcomes. In only one study did the authors examine treated and nontreated women with gestational DM [20]. They reported significant associations with childhood overweight and with obesity in the nontreated group but not in the treated group. Further evidence that treatment of gestational DM may improve offspring outcome is found in studies of women with high blood glucose concentration, but not sufficiently high for a diagnosis of gestational DM thus not treated. For example, Deierlein et al. found that fetal exposure to maternal glucose concentration in the high-normal range was associated with the development of overweight and obesity in the offspring at age three years, independent of maternal prepregnancy BMI [37].

## 8. Future Studies

As previously discussed, most existing observational studies cannot be used to definitively quantify the independent contribution of gestational diabetes to offspring overweight and obesity risk because of unmeasured confounding and other methodological limitations. We recommend the following for future studies.

1. Observational studies should analyze women with pregestational and gestational DM separately, and if possible include documentation of timing of fetal exposure to elevated maternal glucose levels.
2. Observational studies should include documentation of lifestyle and other environmental factors present during infancy and childhood.
3. All studies should address relevant confounders, including maternal prepregnancy BMI. One possible approach would be to make use of databases with linked siblings so outcomes between siblings with discordant exposure to gestational DM could be compared, providing a mechanism to control for shared genetic and lifestyle factors.
4. When possible, randomized trials for the treatment for mild gestational DM should include long-term followup of offspring assess the effects of maternal interventions during pregnancy on infant and child outcomes [38]. If it can be confirmed that treatment of gestational DM is associated with a reduced risk of overweight or obesity in offspring, it will provide further evidence that there is a causal relationship and that an effective prevention intervention exists.

## 9. Limitations

This study has some limitations. First, we did not include unpublished studies, therefore our results may be affected by publication bias. In addition, we may have missed studies that were not listed in PubMed or referenced in other published studies or reviews. Finally, these studies are not all directly comparable due to discrepancies in the study population, methodology, gestational DM diagnostic criteria, BMI reference population, and ages. However, the present study is the first to systematically review all published case-control and cohort studies examining the association between maternal gestational DM and the prevalence of offspring overweight and obesity.

## 10. Conclusions

In conclusion, this review demonstrates that studies of associations between maternal gestational DM on offspring overweight and obesity have yielded inconclusive results. Because maternal obesity is a more prevalent condition than gestational DM and is strongly associated with offspring obesity, we need a better understanding of the relative contributions of maternal obesity and gestational DM to risk before designating infants of women with GDM specifically as targets for obesity prevention efforts. Interventions addressing prepregnancy obesity may have a greater public health impact on childhood overweight and obesity than those targeting offspring of women with gestational DM. A stronger body of evidence is needed to better understand the potential associations between maternal gestational DM and offspring outcomes.

## Figures and Tables

**Table 1:** Studies included in review of maternal gestational diabetes mellitus (GDM) and childhood obesity.

Author, year (population)	Study description (name, years, design)	Number in analysis (cohort)	Child age in years at outcome	GDM diagnosis criteria	Outcome	Number and percent of overweight/obese children among women with and without GDM		Multivariable adjustments (GDM versus no GDM)
						GDM	No GDM	

Boerschmann et al., 2010 (Germany)	German GDM and BABYDIAB study, 1989–2000, prospective	222	11	2 of 3 elevated oral glucose tolerance test of 75 g glucose load	BMI ≥ 90th percentile^d^	23/74 (31.1)	23/148 (15.5)	No

Boney et al., 2005 (USA)	Longitudinal cohort study, years not available, prospective	109 (179)	11	Clinical diagnosis from medical records	BMI > 85th percentile^b^	16/58 (27.6)	14/51 (27.5)	No

Buzinaro et al., 2008 (Brazil)	Hospital cohort,1988–199, prospective	73	10	2 hr clinical measure twice a day in third trimester	BMI > 85th percentile^a^	12/23 (52.2)	4/27 (14.8)	No
					BMI > 95th percentile^a^	1/23 (4.3)	0/27 (0)	

Gillman et al., 2003 (USA)	Nurses Health Study II, 1996, retrospective	14.881 (16.550)	9–14	Maternal self-report from interview/questionnaire	BMI 85th–95th percentile^b^	72/465 (15.5)	1917/14,416 (13.3)	Yes
					BMI > 95th percentile^b^	45/465 (9.7)	958/14,416 (6.6)	

					Carpenter and Coustan:			
					BMI > 85th percentile^b^	60/173 (34.7)	1,788/7,609 (23.5)	
Hillier et al., 2007 (USA)	Kaiser Permanente Hawaii and Northwest, 1995–2000, prospective	7.782 (9.439)	5–7	3 h 100 g oral glucose tolerance test	BMI > 95th percentile^b^	35/173 (20.2)	928/7,609 (12.2)	Yes
					National Diabetes Data Group:			
					BMI > 85th percentile^b^	103/370 (27.8)	1,788/7,609 (23.5)	
					BMI > 95th percentile^b^	64/370 (17.3)	928/7,609 (12.2)	

Lawlor et al., 2010 (United Kingdom)	The Avon Longitudinal Study of Parents and Children (ALSPAC), 1991–1992, prospective	6.584 (10.591)	9–11	Clinical diagnosis from medical records	BMI > 85th percentile^c^	12/40 (30.0)	1481/6544 (22.6)	Yes

Malee et al., 2002 (USA)	Diabetes in Pregnancy Program at the Women and Infants Hospital, 1991–1993, Prospective	64 (262)	9	2 abnormal 100 g glucose tolerance test	BMI ≥ 85th percentile^d^	11/33 (33.3)	8/31 (25.8)	No

Pirkola et al., 2010 (Finland)	Northern Finland 1986 birth cohort, 1985–1986, prospective	745 (4.168)	16	One abnormal value from a 2 hour, 75 g oral glucose tolerance test	BMI > 85th percentile^c^	18/84 (21.4)	113/661 (17.1)	No

Tam et al., 2009 (Hong Kong)	GDM cohort of women at the Prince of Wales Hospital, 1992–1994, prospective	164 (1032)	7–10	75 g oral glucose tolerance test	BMI ≥ 85th percentile^d^	19/63 (30.2)	26/101 (25.5)	No

Whitaker et al., 1998 (USA)	GDM cohort of women from an HMO in Washington state, 1985–1986, prospective	315 (524)	5–10	3 h 100 g oral glucose tolerance test	BMI ≥ 85th percentile^d^	11/58 (19.0)	62/257 (24.1)	No

Wright et al., 2009 (USA)	Project Viva, 1999–2002, prospective	1086 (1.579)	3	Nonfasting oral glucose challenge test	BMI ≥ 95th percentile^b^	7/51 (13.7)	91/1035 (8.8)	No

Wroblewska-Seniuk et al., 2009 (Poland)	Cohort from the Clinical Hospital of Obstetrics and Gynecology in Poznan, Poland, years not available retrospective	185	4–9	Oral glucose tolerance test between 24th and 28th week of gestation	BMI 85th–95th percentile^d^ BMI > 95th percentile^d^	3/34 (8.8) 9/34 (26.5)	8/108 (7.4) 17/108 (15.7)	No

BMI classification reference: ^a^WHO International Classification, ^b^CDC growth charts, ^c^International Obesity Task Force, and ^d^Other.
